# Identification of common differentially expressed genes in Turner (45,X) and Klinefelter (47,XXY) syndromes using bioinformatics analysis

**DOI:** 10.1002/mgg3.1503

**Published:** 2020-09-21

**Authors:** María Carolina Manotas, Juan Camilo Calderón, Liliana López-Kleine, Fernando Suárez-Obando, Olga M. Moreno, Adriana Rojas

**Affiliations:** ^1^ Institute of Human Genetics. Faculty of Medicine Pontificia Universidad Javeriana Bogotá Colombia; ^2^ Department of Statistics Faculty of Science Universidad Nacional de Colombia Ciudad Universitaria Bogotá Colombia

## Abstract

**Background:**

Analysis of patients with chromosomal abnormalities, including Turner syndrome and Klinefelter syndrome, has highlighted the importance of X‐linked gene dosage as a contributing factor for disease susceptibility. Escape from X‐inactivation and X‐linked imprinting can result in transcriptional differences between normal men and women as well as in patients with sex chromosome abnormalities.

**Objective:**

To identify differentially expressed genes among patients with Turner (45,X) and Klinefelter (46,XXY) syndrome using bioinformatics analysis.

**Methodology:**

Two gene expression data sets of Turner (45,X) and Klinefelter syndrome (47,XXY) were obtained from the Gene Omnibus Expression (GEO) database of the National Center for Biotechnology Information (NCBI). Statistical analysis was performed using R Bioconductor libraries. Differentially expressed genes (DEGs) were determined using significance analysis of microarray (SAM). The functional annotation of the DEGs was performed with DAVID v6.8 (The Database for Annotation, Visualization, and Integrated Discovery).

**Results:**

There are no genes over‐expressed simultaneously in both diseases. However, when crossing the list of under‐expressed genes for 45,X cells and the list of over‐expressed genes for 47,XXY cells, there are 16 common genes: SLC25A6, AKAP17A, ASMTL, KDM5C, KDM6A, ATRX, CSF2RA, DHRSX, CD99, ZBED1, EIF1AX, MVB12B, SMC1A, P2RY8, DOCK7, DDX3X, eight of which are involved in the regulation of gene expression by epigenetic mechanisms, regulation of splicing processes and protein synthesis.

**Conclusion:**

Of the 16 identified as under‐expressed in 45,X cells and over‐expressed in 47,XXY cells, 14 are located in X chromosome and 2 in autosomal chromosome; 8 of these genes are involved in the regulation of gene expression: 5 genes are related to epigenetic mechanisms, 2 in regulation of splicing processes, and 1 in the protein synthesis process. Our results are limited by it being the product of a bioinformatic analysis from mRNA isolated from whole blood, this makes necessary further exploration of the relationships between these genes and Turner syndrome and Klinefelter syndrome in the future.

## Graphical abstract

In this manuscript we identify differentially expressed genes among patients with Turner and Klinefelter syndrome. Our results suggest that global epigenetic and RNA expression changes play a central role to the phenotype.

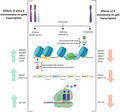

## 1 INTRODUCTION

Turner syndrome (TS) and Klinefelter syndrome (KS) are two of the most common sex chromosome aneuploidies (Berglund et al., 2019). The prevalence of TS is approximately 1 in 2000 to 1 in 4000 among women and is characterized by a chromosomal disorder affecting phenotypically female individuals harboring one intact X chromosome and complete absence [X monosomy (45,X), resulting in 50% of cases] or partial absence of the second X chromosome (including short or long arm deletion, ring X, isochromosome of the long arm and mosaicism, and fusion of 45,X and 46,XX cell lines) along with one or more clinical manifestations (Gravholt et al., 2017; Kesler, 2007). TS presents with broad clinical manifestations, potentially including features such as the characteristic facial appearance (ocular hypertelorism, epicanthal folds, ptosis, a depressed nasal root, short nose with a broad base, low‐set posteriorly rotated ears, and micrognathia), with neck webbing and lymphedema, linear growth failure, ovarian insufficiency (pubertal delay), early sensorineural hearing loss, distinctive congenital cardiovascular, skeletal, digital and renal anomalies, a specific neurodevelopmental profile, and a constellation of other disorders commonly occurring in TS, including hypothyroidism and celiac disease (Gravholt et al., 2017). This broad phenotypic spectrum potentially results from the haploinsufficiency of different genes evading X chromosome inactivation and are normally biallelically expressed in 46, XX women and 46, XY men (Carrel & Willard, 2005; Kesler, 2007; Morgan, 2007).

KS occurs in approximately 1 of 600 newborn boys and is characterized by an extra X chromosome. Most (90%) cases present as a pure form with a 47, XXY karyotype, while the remaining 10% include the following sex‐chromosomal abnormalities: mosaicism (46, XY/47, XXY), higher grade aneuploidy (48, XXXY; 49, XXXXY), and structurally abnormal X chromosomes (Bearelly & Oates, 2019). Men with KS are usually tall, have a gynecoid habitus, are hypogonadal, and may have metabolic, cognitive, psychiatric, and cardiac disorders (Gravholt et al., 2018; Groth, Skakkebæk, Høst, Gravholt, & Bojesen, 2013; Zitzmann et al., 2015) and, similar to women, are 14 times more likely to develop systemic lupus erythematosus (SLE) than 46,XY men (Scofield et al., 2008). However, no distinct dysmorphic features have been reported, and presentation may vary in accordance with the degree of gonadal dysfunction. Furthermore, the severity of disease presentation is strongly correlated with the severity of the sex chromosome aneuploidy, that is, higher‐grade aneuploidies (Bearelly & Oates, 2019).

Although several studies have investigated the association between the karyotype and the severity of the clinical manifestations in TS (Al Alwan, Khadora, & Amir, 2014; Bispo et al., 2013) and a subset of phenotypic characteristics of TS and KS appear to display a linear dose‐dependent association with the number of sex chromosomes, including stature and performance in cognitive subdomains of language and visuospatial processing (Zhang et al., 2020); nevertheless, other phenotypic traits including the altered risk of several autoimmune disorders in both syndromes (Jørgensen et al., 2010; Scofield et al., 2008), cannot exclusively account for sex‐chromosome dosage compensation. In general, the mechanisms through which an extra or absent X chromosome determines the characteristics of KS and TS are poorly understood, and it has been hypothesized that the TS and KS phenotype may result from not only genomic imbalance owing to genes present on sex chromosomes, but also the additive effect on associated genes within a particular gene network with altered gene regulation due to epigenetic factors (Álvarez‐Nava & Lanes, 2018).

Epigenetic mechanisms include all processes involved in the regulation of gene expression. Epigenetic modifications involve the following four mechanisms: (a) DNA methylation of CpG sites in the promoter, (b) posttranslational covalent histone modifications and the use of variant histone proteins, (c) nucleosome remodeling, and (d) regulation of gene expression by noncoding RNA (miRNA and long noncoding RNA). These mechanisms are active during embryonic development, cognitive processing, and lipid and glucose metabolism. However, limited information is available regarding the epigenetic phenomena in TS and KS, since studies on genetic and chromosomal abnormalities explaining the clinical manifestations of these diseases have received increased attention (Álvarez‐Nava & Lanes, 2018).

It is important to understand these regulatory effects to clarify the biological underpinnings of phenotypic sex differences and the clinical features of sex chromosome aneuploidy. This study aimed to compare differentially expressed genes (DEGs) in 45,X and 47, XXY cells. Gene expression patterns were analyzed from microarray data obtained from the National Center for Biotechnology Information (NCBI) Gene Expression Omnibus (GEO) database. Our results show that there are 16 common genes that are oppositely regulated in Turner syndrome (45,X) and in Klinefelter syndrome (47,XXY). It is important to note that eight of these genes are involved in the regulation of gene expression: five genes are related to epigenetic mechanisms, two to regulation of splicing processes and one to the protein synthesis process.

## 2 MATERIALS AND METHODS

We selected two data sets from the NCBI GEO database (accessible at http://www.ncbi.nlm.nih.gov/geo) of microarray data on gene expression obtained from mRNA isolated from whole blood of TS and KS patients. Raw data sets were obtained and processed separately, to render samples comparable in each data set. The preprocessing steps were as follows: quality control and gene sample filtering for non‐correlated or outlier measurements. In house functions and available functions in bioconductor packages were used for these tasks. Furthermore, summarization from probes to genes was carried out for each dataset. Thereafter, we identified DEGs for each data set using bioconductor R packages. Finally, DEGs were compared between both data sets and enrichment analysis was performed.

### 2.1 Selection of the data sets

The TS data set corresponds to the GSE46687 experiment (Cheng, Zhou, & Bondy, 2014) and contains 36 samples, where 10 patients have two X chromosomes (10 controls), 16 patients have an X chromosome of maternal origin, and 10 patients have an X chromosome of paternal origin (26 cases). These data were obtained from the Eunice Kennedy Shriver National Institute of Child Health and Human Development (NICDH) and the US National Institutes of Health (NIH). The data were curated on March 1, 2014. The data update date was March 1, 2014.

The KS data set corresponds to the GSE42331 experiment (Zitzmann, Bongers, & Werler, 2018) and contains 65 samples, where 35 individuals had KS (35 cases) and 30 individuals (15 male, 15 female) were healthy controls. The KS data set only included 47, XXY individuals. These data were curated on July 26, 2018, by Muenster University Hospital, Germany.

Individuals with mosaicism were excluded from both data sets. However, information regarding age and health conditions and their previous medical history was unavailable, potentially constituting a study limitation.

### 2.2 Quality control

Quality control analysis of the two data sets was performed suing descriptive statistics (boxplot, histograms, correlations, and trend statistics) to identify atypical samples and mRNA values. Thereafter, the data were normalized using the *vsn* function (Huber, von Heydebreck, Sültmann, Poustka, & Vingron, 2002) of Bioconductor in R (R Foundation for Statistical Computing, 2018) for each data set separately, and we confirmed that the samples were comparable and that the dependence between the mean and variance was adjusted. We excluded one control sample from the KS data set, since it was an outlier and presented a low correlation with other control samples.

### 2.3 Differential gene expression analysis

DEGs were determined using significance analysis of microarray (SAM) (Tusher, Tibshirani, & Chu, 2001) using the *samr* package. At each time point, DEGs were detected by comparing the mean gene expression levels under the two conditions (controls vs. TS/KS). Differential gene expression analysis was performed individually for each data set. This analysis yielded lists of DEGs between control and TS/KS individuals.

SAM was performed to detect DEGs by assigning a score (analogue to the *t* statistic, called di) to each gene in accordance with the mean expression levels relative to the standard deviation of repeated measures of each condition. To determine whether this score is greater than a specified threshold, the probability (*p*‐value) was determined through permutations of repeated measures to generate a probability distribution. Furthermore, the percentage of genes potentially exceeding this threshold simply owing to chance events was determined and reported as the false discovery rate (FDR), facilitating double‐filtering of false‐positive results. The user can use a desired FDR percentage reflecting the confidence levels of DEGs detected therein.

### 2.4 Gene set enrichment analysis

DEG lists were annotated and enriched using DAVID tools (Huang, Sherman, & Lempicki, 2009), wherein abnormal annotation terms were identified in the gene list by comparing its frequency to the expected frequency through chance events. This procedure was carried out for each annotation term, using a hypothesis test including the chi‐square or Fisher's exact test, and the *p*‐value was then corrected for multiple testing.

## 3 RESULTS

The TS data set used the reference human genome HG‐U133_Plus_2, thus, facilitating effective summarization in R and differential gene expression analysis at the gene level. However, the KS data set used the reference human genome HuGene‐1_0‐st, which resulted in complications during summarization, since numerous probes in the R library do not correspond to any gene. Therefore, for KS, differential gene expression analysis was performed at the probe level and the probes were annotated to identify the gene they correspond to, using the DAVID notation tool (Jiao et al., 2012).

**FIGURE 1:**
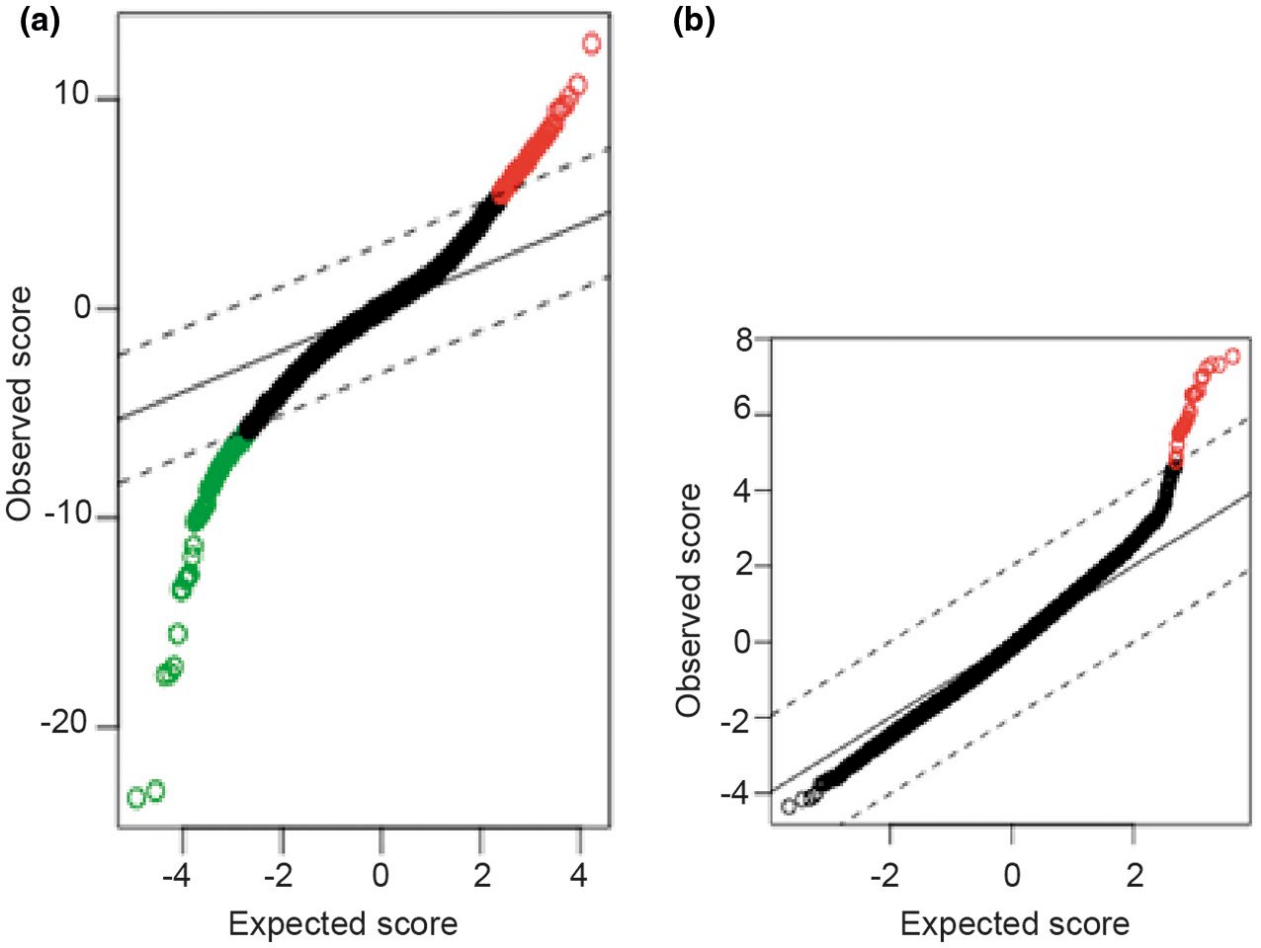
The SAM plot of overexpressed and downregulated genes. (a) The Turner's syndrome SAM plot given a d_i_ = 3.0649, differentially expressed genes deviate from the diagonal area: upregulated genes are colored in red dots in the upright corner and green dots in the bottom‐left corner represent downregulated. (b) The Klinefelter's syndrome SAM plot given a d_i_ = 0.9579, differentially expressed genes deviate from the diagonal area: upregulated genes are colored in red dot in the upright corner, not observed downregulated genes

For TS, a critical value of di = 3.0649 was selected, corresponding to a false positive rate approaching zero (FDR < 2E16). In total, 996 DEGs were identified, of which 501 were upregulated and 495 were downregulated (Figure 1). Similarly, a value of di = 0.9579 (FDR = 0.0770) was determined for KS, thus, retaining the false‐positive ratio at zero. Since differential gene expression analysis for KS was performed at the probe level, all differentially expressed probes were selected and, through DAVID enrichment analysis (Jiao et al., 2012), were associated with a gene. Finally, 56 genes were over‐expressed (Figure 1).

**FIGURE 2:**
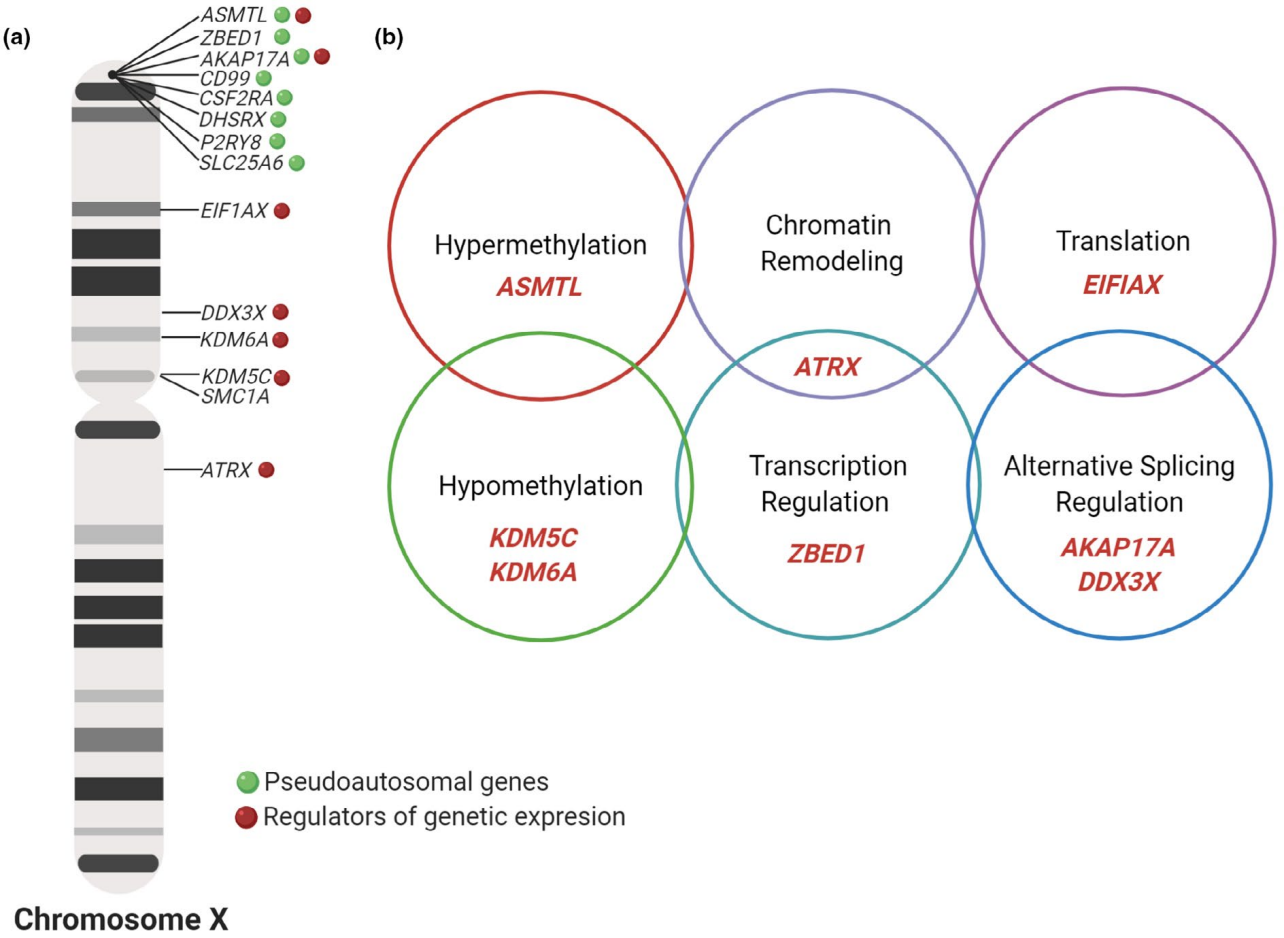
X‐linked genes upregulated in Klinefelter Syndrome and downregulated in Turner Syndrome. Differential patterns of gene expression obtained from microarray data from the National Center for Biotechnology Information (NCBI) Gene Expression Omnibus (GEO) database (a). Presence of 14 common genes on the X chromosome, which are oppositely regulated in Turner syndrome and in Klinefelter syndrome. Pseudoautosomal genes are represented in green and genes coding for gene expression regulators are represented in red. (b) 8 genes involved in the regulation of gene expression: five genes are related to epigenetic mechanisms, two to regulation of splicing processes and one to the protein synthesis process. Include highest earned academic degree for authors on title page. Created with BioRe​nder.com

To identify DEGs common to TS and KS, we initially searched for matches between the lists of upregulated genes; however, no genes were simultaneously upregulated in TS and KS. However, on cross‐checking the list of downregulated genes for TS and upregulated genes for KS, 16 genes overlapped (Table 1). In particular, most genes displaying changes in their expression levels were located on the X chromosome, except for *DOCK7* and *MVB12B*, which are located at 1p31.3 and 9q33.33, respectively (Table 1) (Figure 2a).

**TABLE 1:** Genes that are downregulated in Turner syndrome and upregulated in Klinefelter syndrome

Gene	Protein	Location	Related function
*DDX3X*	DEAD‐box helicase 3 X‐linked	Xp11.4	Transcriptional regulation‐splicing and export of mRNA
*ASMTL*	Acetylserotonin O‐Methyltransferase Like	Xp22.33/Yp11.2	Methyltransferase activity
*ATRX*	ATRX, chromatin remodeler	Xq21.1	Transcriptional regulation and chromatin remodeling
*EIF1AX*	Eukaryotic Translation Initiation Factor 1A X‐Linked	Xp22.12	Participates in translation. Stabilizes the binding of tRNA‐met to the ribosomal subunit 40 s.
*KDM5C*	Lysine Demethylase 5C	Xp11.22	Demethylates lys‐4 of Histone 3. Participates in the transcriptional repression of neuronal genes. Represses the expression of neuronal genes.
*KDM6A*	Lysine Demethylase 6A	Xp11.3	Called UTX. Demethylates lys‐27 of Histone 3. Regulates the expression of the HOX genes.
*ZBED1*	Zinc Finger BED‐Type Containing 1	Xp22.33/Yp11.2	Stimulates the transcription of Histone 1.
*AKAP17A*	A‐Kinase Anchoring Protein 17A	Xp22.33/Yp11.2	Regulator of the alternative splicing
*CD99*	CD99 antigen	Xp22.33/Yp11.2	Cell adhesion, migration, death, differentiation, and diapedesis
*CSF2RA*	Colony stimulating Factor 2 Receptor Subunit Alpha	Xp22.33/Yp11.2	Proliferation, differentiation, and functional activation of hematopoietic cells
*DOCK7*	Dedicator of cytokinesis 7	1p31.3	Axon formation and neuronal polarization. Cortical neurogenesis (glial cell proliferation) Negatively regulates apical‐basal interkinetic nuclear migration of radial glial cells
*DHRSX*	Dehydrogenase/Reductase X‐Linked	Xp22.33/Yp11.2	Positive regulation of starvation‐induced autophagy
*P2RY8*	P2Y Receptor Family Member 8		G‐protein‐coupled receptor that responds to purine and pyrimidine nucleotides
*SLC25A6*	Solute Carrier Family 25 Member 6	Xp22.33/Yp11.2	Cytoplasmic ADP exchange with mitochondrial ATP. Formation of the permeability transition pore involved in apoptosis
*SMC1A*	Structural Maintenance Of Chromosomes 1, Yeast	Xp11.22	Chromosomal cohesion during the cell cycle and DNA repair
*MVB12B*	Multivesicular Body Subunit 12B	9q33.3	Endocytosis regulator

## 4 DISCUSSION

X chromosome inactivation (XCI), dysregulation of gene dosage and the parental origin of the X chromosome are the phenotypic determinants of TS and KS (Trolle et al., 2016; Viana et al., 2014). Through in silico analysis of microarray data, this study reports 16 genes downregulated in TS and upregulated in KS, including *KDM5C*, *KDM6A*, *ZBED1*, *ASMTL*, *ATRX*, *DDX3X*, *AKAP17A*, *EIF1AX*, *CD99*, *CSF2RA*, *DOCK7*, *DHRSX*, *P2RY8*, *SLC25A6*, *SMC1A*, and *MVB12B* (Table 1). Twelve of these 16 genes were upregulated in KS patients according to the original data of data set GSE42331 analyzed by Zitzmann et al., 2015, and *ATRX*, *AKAP17A*, *DHRSX*, and the autosomal gene *MVB12B* were not reported to be upregulated. This difference may have been obtained because in their study, transcriptome profiling was restricted to only two individuals, while herein, all data corresponding to 65 samples are described.

Fourteen of these 16 DEGs between TS and KS are located on the X chromosome, 8 of which (*ZBED1*, *ASMTL*, *AKAP17A*, *CD99*, *CSF2RA*, *DHRSX*, *P2RY8*, and *SMC1A*) are located in the pseudo‐autosomal 1 region of the X or Y chromosome (*PAR1*) (Figure 2a), owing to the presence of the extra or missing X chromosome, characteristic of KS and TS. This was expected, considering the canonical model for sex chromosome dosage compensation (SCD), which states that PAR genes are potentially upregulated with an increase in the X or Y chromosome count, while Y‐linked genes are upregulated linearly with an increase in the Y chromosome count. Owing to the non‐binary nature of XCI‐mediated gene silencing, the theorized effects of SCD on SCI‐related gene expression and X‐linked genes evading XCI (XCIE) represent the extreme ends of an X‐chromosome dosage‐sensitivity continuum. A recent study reported that an X‐linked gene completely silenced on XCI displays an inverse correlation between significant XCI cluster expression and X‐chromosome dosage, whereas an X‐linked gene completely evading XCI displays sublinear upregulation with an increase in the X chromosome count (Raznahan et al., 2018).

**FIGURE 3:**
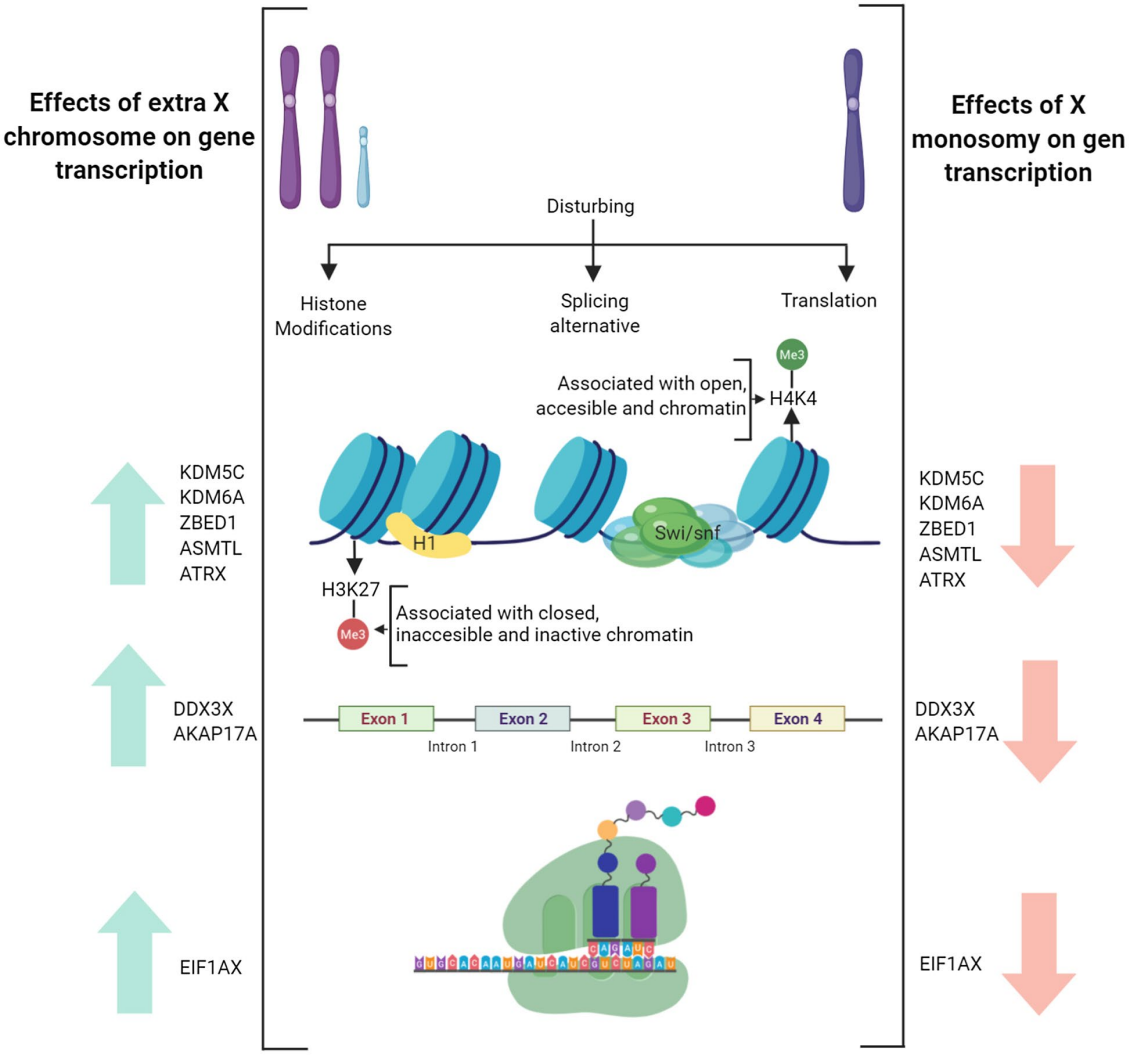
Differentially expressed genes in Turner syndrome and Klinefelter syndrome involved in gene expression mechanisms. Proposed model showing eight genes expressed differentially in TS and KS. KDM5C, KDM6A, ZBED1, ASMTL, and ATRX genes are involved in epigenetic processes. DDX3X and AKAP17A genes encode for regulatory proteins of splicing processes, and EIFIAX is related to the protein synthesis process. Created with BioRe​nder.com

Interestingly, 8 of the 16 genes encode proteins regulating gene through different mechanisms, including epigenetic regulators (*KDM5C*, *KDM6A*, *ASMTL*, *ATRX*, and *ZBED1*), splicing regulators (*DDX3X* and *AKAP17A*), and translation regulators (*EIF1AX*) (Figure 3). These genes are reportedly upregulated in KS patients in comparison with 46,XY individuals (Skakkebæk et al., 2018), which in addition to validating our results, suggests other causes for these pathologies, considering the gene sequence or the overall impact of chromosomal imbalance (Álvarez‐Nava & Lanes, 2018) (Table 1). *KDM5C*, *KDM6A*, *EIF1AX*, and *DDX3X* evade XCI (Figure 2a) (Fang, Disteche, & Berletch, 2019; Y. Zhang et al., 2013) and a previous study investigating sex chromosome dosage through the analysis of genome‐wide expression data in humans with diverse sex chromosome aneuploidies (XO, XXX, XXY, XYY, and XXYY), disparities in X chromosome dosage were always accompanied by significant differences in expression levels in the aforementioned four genes (Raznahan et al., 2018).

KDM5C functions as an enzyme erasing the activating H4K4Me3 signature and plays a role in cognition, resulting in the neurocognitive phenotype observed among KS patients (Skakkebæk et al., 2018). However, *KDM6A* encodes demethylase UTX, which erases the repressing H3K27Me3 signature (Allis, Caparros, Jenuwein, & Reinberg, 2015), whose expression levels and the levels of EIF1AX, DDX3X, and CD99 are associated with an increased abdominal circumference among KS patients and with serum IL‐6, TNF, C‐reactive protein, and procoagulant plasminogen activator inhibitor 1 (PAI‐1) levels, which are potentially associated with an increased risk of metabolic syndrome among KS patients (Zitzmann et al., 2015).

In TS, KDM6A haploinsufficiency is associated with gonadal dysgenesis associated with this disease (Berletch, Deng, Nguyen, & Disteche, 2013). Abnormal changes in *KDM5C* and *KDM6A* expression levels lead to patterns of aberrant histone modifications potentially affecting transcriptional regulation in different cellular phenomena (Allis et al., 2015) (Figure 3).

Regarding the other genes located in PAR1, including *ZBED1* and *ASMTL*, *ZBED1* encodes a transcription factor that regulates the activation of H1 (a linker histone mediating chromatin stability) promoters (Figure 3). Because of its biallelic expression, the gain or loss of an X chromosome can affect its expression and function, similar to *ASMTL*, which encodes N‐acetylserotonin O‐methyltransferase‐like protein (Allis et al., 2015). Otherwise, *ATRX* encodes a transcriptional regulator with an ATPase/helicase domain and belongs to the SWI/SNF family of chromatin remodeling proteins, being required for the deposition of the histone variant H3.3 at telomeres and other genomic repeats. These interactions are important to retain silencing marks at these sites (Allis et al., 2015). *DDX3X* and *AKAP17A* encode splicing regulators (Figure 3); hence, alterations in their expression potentially affects their downstream protein‐coding genes (Alberts, 2007). Furthermore, *EIF1AX* encodes an essential eukaryotic translation initiation factor, which is a component of the 43S pre‐initiation complex (PIC), which mediates the recruitment of the small 40S ribosomal subunit to the 5′ cap of mRNAs (Figure 2b) (Alberts, 2007). These findings support those of previous studies reporting numerous quantitative changes in gene expression in TS and KS (Allis et al., 2015). Moreover, apart from simple transcriptional effects, a previous hypothesis suggesting that the presence or absence of X chromosomes act in *trans* to elicit histotypic modifications in histones, DNA methylation signatures, and the chromatin landscape at genome‐wide distributed loci, thus potentially elucidating the pathogenesis of TS and LS from the epigenetic viewpoint (Álvarez‐Nava & Lanes, 2018) (Figure 3).

Regarding *CD99* and *CSF2RA* located in PAR1, a recent study using the same data set used herein GSE46687), investigating DEGs between X monosomy TS patients and healthy women, reported that *CD99* and *CSF2RA* were downregulated, thus, potentially increasing the frequency of autoimmune diseases among women with TS (Wang et al., 2020). This study shows that *CD99* and *CSF2RA* are potentially associated with the pathogenesis of TS, although our study was different and describes the comparison of extreme phenotypes (45,X and 47, XXY cells).

CD99 contributes to the pathogenesis of systemic lupus erythematosus (SLE) (Fattal et al., 2010). SLE is more prevalent among women, and men with KS (47, XXY men) are at a similar risk of SLE as women (Scofield et al., 2008)(Cooney et al., 2009). Moreover, TS patients are at an increased risk of autoimmune diseases, most notably autoimmune thyroid diseases and type 1 diabetes mellitus, and are at a lower risk of SLE (Jørgensen et al., 2010). These findings concurrently suggest a gene dose effect at the X chromosome, indicating that individuals with two X chromosomes (46,XX or 47,XXY), are at a higher risk of SLE than those with one X chromosome (46,XY or 45,X) (Cooney et al., 2009), potentially consistent with the dose of *CD99*.

A histotypic expression pattern of *SLC25A6* and *DHRSX*, located in PAR1, has been previously reported among KS patients. *SLC25A6* is reportedly overexpressed in peripheral blood and cerebellar leukocytes among KS patients (Skakkebæk et al., 2018; Viana et al., 2014), *DHRSX* is overexpressed in the cerebellum and the prefrontal cortex of the same individual (Viana et al., 2014). However, the association between the potential role of *SLC25A6* and *DHRSX* in the neurocognitive phenotype in KS and TS remains unknown. *SLC25A6* upregulation is reportedly associated with a shorter QTc interval in KS patients (Zitzmann et al., 2015). *CSF2RA* is also located in PAR1 and its upregulation in KS is associated with insulin resistance, waist circumference, and levels of pro‐coagulatory substance PAI‐1 and cytokines (Ross, Roeltgen, Kushner, Wei, & Zinn, 2000). *CSF2RA* haploinsufficiency is associated with a short stature among TS patients (Joseph et al., 1996).

Furthermore, herein, two autosomal genes including *DOCK7* and *MVB12B* were up‐regulated in KS and down‐regulated in TS. *DOCK7* positively regulates gene expression among KS patients (Skakkebæk et al., 2018; Zitzmann et al., 2015). Furthermore, DOCK7 is implicated in the neurocognitive phenotype in KS (Skakkebæk et al., 2018), considering its contribution to the cartridge/bouton, synapse development, and chandelier cell (ChC) morphogenesis; ChCs are a unique subset of GABAergic interneurons that selectively innervate the axon initial segment of excitatory pyramidal neurons (Gallo, Paul, & Aelst, 2020). However, a previous study, with an approach similar to the present study, reported that DOCK7 and MVB12B were differentially expressed in KS and TS (Zhang et al., 2020). MVB12B is associated with endocytosis and innate immunity (Nandakumar et al., 2019); however, its potential association with the pathophysiology of KS and TS is unknown. Genetic interactions between these and specific genes present on sex chromosomes remain unclear; however, this is potentially conditioned by the aberrant expression of some genes identified herein, which encode transcriptional regulators. Moreover, loss of sex chromosome material affects autosomal gene expression (Trolle et al., 2016), and sex chromosomes globally regulate gene expression, with an approximate 3% influence on autosomal genes (Trolle et al., 2016). Furthermore, the potential of SCD to disrupt genome function extensively varies, indicating that differential involvement of autosomal genes is central to this variation (Raznahan et al., 2018).

Our results indicate that genes present in PARs in sex chromosomes, particularly including *ASMTL*, *ZBED1*, *AKAP17A*, *CD99*, *CSF2RA*, *DHRSX*, and *SLC25A6*, and those evading XCI, including *KDM5C*, *KDM6A*, *EIF1AX*, and *DDX3X*, which are biallelically expressed under normal conditions, potentially account for some clinical features of TS and KS. Furthermore, 8 of the 16 genes, overlapping and displaying opposite expression profiles in TS and KS, encoded transcriptional regulators, thus greatly influencing embryonic development (Figure 3).

Our study has several limitations. Being an in silico study, and notwithstanding previous studies on blood mRNA profiling yielding indicators for different diseases (Olsen, Skeie, & Lund, 2015), the tissue‐level relevance of the present findings might be challenged. Our results may guide further studies on the role of *KDM5C*, *KDM6A*, *ZBED1*, *ASMTL*, *ATRX*, *DDX3X*, *AKAP17A*, *EIF1AX*, *CD99*, *CSF2RA*, *DOCK7*, *DHRSX*, *P2RY8*, *SLC25A6*, *SMC1A*, and *MVB12B* in the pathophysiology of TS and KS. Furthermore, DNA methylation and gene expression assays for phenotypes observed with karyotypes 47,XXY and 45,X in different tissues and functional validation of the genes reported herein would further support the present findings and elucidate the role of these genes in the phenotypes of TS and KS, respectively.

## CONFLICT OF INTEREST

None declared.

## AUTHOR’S CONTRIBUTIONS

MM, JC performed the bioinformatics analysis and drafted the manuscript. FS and OM clinical analysis, manuscript preparation. LL and AR conceived the experimental and bioinformatics plan, data analysis, project coordination, supervised the project.

## References

[mgg31503-bib-0001] Al Alwan, I. , Khadora, M. , & Amir, I. (2014). Turner Syndrome Genotype and phenotype and their effect on presenting features and timing of Diagnosis. International Journal of Health Sciences, 8(2), 195–202. 10.12816/0006086 25246887 PMC4166992

[mgg31503-bib-0002] Alberts, B. (2007). Molecular biology of the cell (5th ed.). New York 10.1249/mss.0b013e318185ce9d

[mgg31503-bib-0003] Allis, D.C. , Caparros, M.-L. , Jenuwein, T. , & Reinberg, D. (2015). Epigenetics (2nd ed.). New York Retrieved from https://cshlp​ress.com/defau​lt.tpl?cart=15832​87911​98326​66&actio​n=full&--eqsku​datar​q=987

[mgg31503-bib-0004] Álvarez‐Nava, F. , & Lanes, R. (2018). Epigenetics in Turner syndrome. Clinical Epigenetics, 10(45), 1–20. 10.1186/s13148-018-0477-0 29636833 PMC5889574

[mgg31503-bib-0005] Bearelly, P. , & Oates, R. (2019). Recent advances in managing and understanding Klinefelter syndrome. F1000Research, 8, 112 10.12688/f1000research.16747.1 PMC6352920 30755791

[mgg31503-bib-0006] Berglund, A. , Viuff, M.H. , Skakkebæk, A. , Chang, S. , Stochholm, K. , & Gravholt, C.H. (2019). Changes in the cohort composition of turner syndrome and severe non‐diagnosis of Klinefelter, 47, XXX and 47, XYY syndrome: a nationwide cohort study. Orphanet Journal of Rare Diseases, 14(1), 16 10.1186/s13023-018-0976-2 30642344 PMC6332849

[mgg31503-bib-0007] Berletch, J.B. , Deng, X. , Nguyen, D.K. , & Disteche, C.M. (2013). Female bias in Rhox6 and 9 regulation by the histone demethylase KDM6A. PLoS Genetics, 9(5), e1003489 10.1371/journal.pgen.1003489 23658530 PMC3642083

[mgg31503-bib-0008] Bispo, A. , dos Santos, L.O. , Burégio‐Frota, P. , Galdino, M.B. , Duarte, A.R. , Leal, G.F. , … Santos, N. (2013). Effect of chromosome constitution variations on the expression of Turner phenotype. Genetics and Molecular Research: GMR, 12(4), 4243–4250. 10.4238/2013.March.13.13 23546984

[mgg31503-bib-0009] Carrel, L. , & Willard, H.F. (2005). X‐inactivation profile reveals extensive variability in X‐linked gene expression in females. Nature, 434(7031), 400–404. 10.1038/nature03479 15772666

[mgg31503-bib-0010] Cheng, C. , Zhou, J. , & Bondy, C. (2014).Gene Expression Profiling in 45X Turner Syndrome patients. Retrieved from https://www.ncbi.nlm.nih.gov/geo/query/​acc.cgi?acc=GSE46687

[mgg31503-bib-0011] Cooney, C.M. , Bruner, G.R. , Aberle, T. , Namjou‐Khales, B. , Myers, L.K. , Feo, L. , … Scofield, R.H. (2009). 46, X, del(X)(q13) Turner’s syndrome women with systemic lupus erythematosus in a pedigree multiplex for SLE. Genes and Immunity, 10(5), 478–481. 10.1038/gene.2009.37 19458623 PMC2722751

[mgg31503-bib-0012] Fang, H. , Disteche, C.M. , & Berletch, J.B. (2019). X inactivation and escape: Epigenetic and structural features. Frontiers in Cell and Developmental Biology, 7, 1–12. 10.3389/fcell.2019.00219 31632970 PMC6779695

[mgg31503-bib-0013] Fattal, I. , Shental, N. , Mevorach, D. , Anaya, J.-M. , Livneh, A. , Langevitz, P. , … Cohen, I.R. (2010). An antibody profile of systemic lupus erythematosus detected by antigen microarray. Immunology, 130(3), 337–343. 10.1111/j.1365-2567.2010.03245.x 20201986 PMC2913213

[mgg31503-bib-0014] Gallo, N.B. , Paul, A. , & Aelst, L.V. (2020). Shedding light on chandelier cell development, connectivity, and contribution to neural disorders. Trends in Neurosciences, 43, 565–580. 10.1016/j.tins.2020.05.003 32564887 PMC7392791

[mgg31503-bib-0015] Gravholt, C.H. , Andersen, N.H. , Conway, G.S. , Dekkers, O.M. , Geffner, M.E. , Klein, K.O. , … Sandberg, D.E. (2017). Clinical practice guidelines for the care of girls and women with Turner syndrome: Proceedings from the 2016 Cincinnati International Turner Syndrome Meeting. European Journal of Endocrinology, 177(3), G1–G70. 10.1530/EJE-17-0430 28705803

[mgg31503-bib-0016] Gravholt, C.H. , Chang, S. , Wallentin, M. , Fedder, J. , Moore, P. , & Skakkebæk, A. (2018). Klinefelter syndrome: Integrating genetics, neuropsychology, and endocrinology. Endocrine Reviews, 39(4), 389–423. 10.1210/er.2017-00212 29438472

[mgg31503-bib-0017] Groth, K.A. , Skakkebæk, A. , Høst, C. , Gravholt, C.H. , & Bojesen, A. (2013). Klinefelter syndrome—A clinical update. The Journal of Clinical Endocrinology & Metabolism, 98(1), 20–30. 10.1210/jc.2012-2382 23118429

[mgg31503-bib-0018] Huang, D.W. , Sherman, B.T. , & Lempicki, R.A. (2009). Systematic and integrative analysis of large gene lists using DAVID bioinformatics resources. Nature Protocols, 4(1), 44–57. 10.1038/nprot.2008.211 19131956

[mgg31503-bib-0019] Huber, W. , von Heydebreck, A. , Sültmann, H. , Poustka, A. , & Vingron, M. (2002). Variance stabilization applied to microarray data calibration and to the quantification of differential expression. Bioinformatics (Oxford, England), 18(Suppl 1), S96–S104. 10.1093/bioinformatics/18.suppl_1.s96 12169536

[mgg31503-bib-0020] Jiao, X. , Sherman, B.T. , Huang, D.W. , Stephens, R. , Baseler, M.W. , Lane, H.C. , & Lempicki, R.A. (2012). DAVID‐WS: a stateful web service to facilitate gene/protein list analysis. Bioinformatics (Oxford, England), 28(13), 1805–1806. 10.1093/bioinformatics/bts251 PMC3381967 22543366

[mgg31503-bib-0021] Jørgensen, K.T. , Rostgaard, K. , Bache, I. , Biggar, R.J. , Nielsen, N.M. , Tommerup, N. , & Frisch, M. (2010). Autoimmune diseases in women with Turner’s Syndrome. Arthritis & Rheumatism, 62(3), 658–666. 10.1002/art.27270 20187158

[mgg31503-bib-0022] Joseph, M. , Cantú, E.S. , Pai, G.S. , Willi, S.M. , Papenhausen, P.R. , & Weiss, L. (1996). Xp pseudoautosomal gene haploinsufficiency and linear growth deficiency in three girls with chromosome Xp22;Yq11 translocation. Journal of Medical Genetics, 33(11), 906–911. 10.1136/jmg.33.11.906 8950669 PMC1050783

[mgg31503-bib-0023] Kesler, S.R. (2007). Turner syndrome. Child and Adolescent Psychiatric Clinics of North America, 16(3), 709–722. 10.1016/j.chc.2007.02.004 17562588 PMC2023872

[mgg31503-bib-0024] Morgan, T. (2007). Turner syndrome: Diagnosis and management. American Family Physician, 76(3), 405–417. Retrieved from https://www.aafp.org/afp/2007/0801/p405.html 17708142

[mgg31503-bib-0025] Nandakumar, R. , Tschismarov, R. , Meissner, F. , Prabakaran, T. , Krissanaprasit, A. , Farahani, E. , … Paludan, S.R. (2019). Intracellular bacteria engage a STING‐TBK1-MVB12b pathway to enable paracrine cGAS‐STING signalling. Nature Microbiology, 4(4), 701–713. 10.1038/s41564-019-0367-z PMC6433288 30804548

[mgg31503-bib-0026] Olsen, K.S. , Skeie, G. , & Lund, E. (2015). Whole‐blood gene expression profiles in large‐scale epidemiological studies: What do they tell? Current Nutrition Reports, 4(4), 377–386. 10.1007/s13668-015-0143-5 26568898 PMC4639574

[mgg31503-bib-0027] R Foundation for Statistical Computing . (2018). R: A language and environment for statistical computing, Vienna, Austria: Foundation for Statistical Computing Retrieved from http://www.R‐proje​ct.org/

[mgg31503-bib-0028] Raznahan, A. , Parikshak, N.N. , Chandran, V. , Blumenthal, J.D. , Clasen, L.S. , Alexander‐Bloch, A.F. , … Geschwind, D.H. (2018). Sex‐chromosome dosage effects on gene expression in humans. Proceedings of the National Academy of Sciences of the United States of America, 115(28), 7398–7403. 10.1073/pnas.1802889115 29946024 PMC6048519

[mgg31503-bib-0029] Ross, J.L. , Roeltgen, D. , Kushner, H. , Wei, F. , & Zinn, A.R. (2000). The turner syndrome‐associated neurocognitive phenotype maps to distal Xp. American Journal of Human Genetics, 67(3), 672–681. 10.1086/303039 10931762 PMC1287527

[mgg31503-bib-0030] Scofield, R.H. , Bruner, G.R. , Namjou, B. , Kimberly, R.P. , Ramsey‐Goldman, R. , Petri, M. , … Harley, J.B. (2008). Klinefelter’s syndrome (47, XXY) in male systemic lupus erythematosus patients: Support for the notion of a gene‐dose effect from the X chromosome. Arthritis and Rheumatism, 58(8), 2511–2517. 10.1002/art.23701 18668569 PMC2824898

[mgg31503-bib-0031] Skakkebæk, A. , Nielsen, M.M. , Trolle, C. , Vang, S. , Hornshøj, H. , Hedegaard, J. , … Gravholt, C.H. (2018). DNA hypermethylation and differential gene expression associated with Klinefelter syndrome. Scientific Reports, 8(1), 13740 10.1038/s41598-018-31780-0 30213969 PMC6137224

[mgg31503-bib-0032] Trolle, C. , Nielsen, M.M. , Skakkebæk, A. , Lamy, P. , Vang, S. , Hedegaard, J. , … Gravholt, C.H. (2016). Widespread DNA hypomethylation and differential gene expression in Turner syndrome. Scientific Reports, 6(1), 34220 10.1038/srep34220 27687697 PMC5043230

[mgg31503-bib-0033] Tusher, V.G. , Tibshirani, R. , & Chu, G. (2001). Significance analysis of microarrays applied to the ionizing radiation response. Proceedings of the National Academy of Sciences of the United States of America, 98(9), 5116–5121. 10.1073/pnas.091062498 11309499 PMC33173

[mgg31503-bib-0034] Viana, J. , Pidsley, R. , Troakes, C. , Spiers, H. , Wong, C.C.Y. , Al‐Sarraj, S. , … Mill, J. (2014). Epigenomic and transcriptomic signatures of a Klinefelter syndrome (47, XXY) karyotype in the brain. Epigenetics, 9(4), 587–599. 10.4161/epi.27806 24476718 PMC4121369

[mgg31503-bib-0035] Wang, H. , Zhu, H. , Zhu, W. , Xu, Y. , Wang, N. , Han, B. , … Qiao, J. (2020). Bioinformatic analysis identifies potential key genes in the pathogenesis of Turner syndrome. Frontiers in Endocrinology, 11, 104 10.3389/fendo.2020.00104 32210915 PMC7069359

[mgg31503-bib-0036] Zhang, X. , Hong, D. , Ma, S. , Ward, T. , Ho, M. , Pattni, R. , … Urban, A.E. (2020). Integrated functional genomic analyses of Klinefelter and Turner syndromes reveal global network effects of altered X chromosome dosage. Proceedings of the National Academy of Sciences, 117(9), 4864–4873. 10.1073/pnas.1910003117 PMC7060706 32071206

[mgg31503-bib-0037] Zhang, Y. , Castillo‐Morales, A. , Jiang, M. , Zhu, Y. , Hu, L. , Urrutia, A.O. , … Hurst, L.D. (2013). Genes that escape X‐inactivation in humans have high intraspecific variability in expression, are associated with mental impairment but are not slow evolving. Molecular Biology and Evolution, 30(12), 2588–2601. 10.1093/molbev/mst148 24023392 PMC3840307

[mgg31503-bib-0038] Zitzmann, M. , Bongers, R. , Werler, S. , Bogdanova, N. , Wistuba, J. , Kliesch, S. , … Tüttelmann, F. (2018).Gene expression data from whole blood of Klinefelter Syndrome patients compared to male and female controls. Retrieved from https://www.ncbi.nlm.nih.gov/geo/query/​acc.cgi?acc=GSE42331

[mgg31503-bib-0039] Zitzmann, M. , Bongers, R. , Werler, S. , Bogdanova, N. , Wistuba, J. , Kliesch, S. , … Tüttelmann, F. (2015). Gene expression patterns in relation to the clinical phenotype in Klinefelter syndrome. The Journal of Clinical Endocrinology & Metabolism, 100(3), E518–E523. 10.1210/jc.2014-2780 25532039

